# Inadvertent insertion of a subclavian central venous catheter into the pulmonary artery through a Blalock-Taussig shunt: a rare complication of a common procedure

**DOI:** 10.1186/s13019-025-03638-w

**Published:** 2025-11-03

**Authors:** Mehdi Ghaderian, Mohadeseh Ghasemi

**Affiliations:** Pediatric Cardiovascular Research Center, Cardiovascular Research Institute, Isfahan University of Medical Sciences, Isfahan, Iran

**Keywords:** Blalock-Taussig procedure, Central venous catheters, Malpositioned central venous catheter, Congenital heart disease

## Abstract

Central venous catheter (CVC) insertion is a common procedure providing direct access to the central venous system. Potential mechanical, infectious, and embolic complications could occur. Malpositioning into the pulmonary artery is a very rare complication. A modified Blalock-Taussig shunt (MBTS), anatomically positioned between the subclavian and the pulmonary arteries and adjacent to the right internal jugular vein, is the most common procedure performed in children with complex congenital heart disease (CHD) to enhance pulmonary artery blood flow. Here, we present an infant with a complex CHD and an MBTS in whom a CVC was placed under ultrasound guidance. The misplacement of the CVC was suspected due to deviation of the CVC in chest radiography, elevated PvO2 levels obtained from the CVC, and an increased central venous pressure recorded via the CVC. Cardiac catheterization confirmed that the CVC tip was located in the pulmonary artery, having been inserted through MBTS. The catheter likely traversed the right carotid artery, subclavian artery, Blalock-Taussig (BT) shunt, and pulmonary artery.

## Introduction

Central venous catheters (CVCs), also called central venous access devices, provide long-term venous access to administer parenteral nutrition, blood products, and complex intravenous medicines [1]. These devices are widely used worldwide, especially in ICU patients. It has been estimated that more than 5 million CVCs are being inserted in the United States each year [2]. CVCs are inserted into the central venous vasculature, lying within the proximal third of the superior vena cava, the right atrium, or the inferior vena cava, and being inserted through a proximal central vein, most commonly the subclavian, internal jugular, or femoral vein or a peripheral vein, through an invasive procedure. As an invasive procedure, CVC insertion is associated with potential complications, rating between 0.4% and 20%, classifying into immediate and delayed, which are secondarily subdivided into mechanical, embolic, and infectious [3, 6]. Mechanical complications of CVC insertion are associated with the procedure of CVC placement itself, the frequency of which varies according to physician experience, the site of CVC insertion, and the use of ultrasound guidance. Inadvertent insertion of a CVC into pulmonary arteries as a very rare complication is only reported in some case reports and was not listed in complication lists in previous studies [4]. The modified-Blalock-Taussig Shunt (MBTS) is a common cardiovascular device used to improve pulmonary blood flow in patients suffering from congenital heart disease (CHD) causing impeded pulmonary blood flow [5, 8]. In this study, we report a patient in whom a CVC was inadvertently inserted into the pulmonary artery through an MBTS. Its malposition was suspected through abnormal venous blood gas (VBG) test results, which were confirmed by angiography.

## Case presentation

A full-term (37 weeks) 2.5 kg male infant who was born by cesarean section was cyanotic at birth with decreased oxygen saturation levels. Initial discharge at 20 days had a saturation of approximately 84%. Further evaluation with echocardiography confirmed pulmonary atresia, large subaortic ventricular septal defect (VSD), patent ductus arteriosus (PDA), and overriding of the aorta. After 20 days of hospitalization, he was discharged without further procedures.

On day 55 of life, he was hospitalized following decreased blood oxygen saturation, with saturation declining to 65–70%. On day 63 of life, he underwent a right MBTS shunt insertion (size: No. 5). Post-shunt, saturation improved to 87–88%.

Twenty days later, during hospitalization, on day 83 of life, he was diagnosed with sepsis and subsequently developed apnea and severe acidosis. Because of the patient’s critically ill condition and difficult peripheral venous access, a CVC was inserted under real-time ultrasound guidance. There wasn’t a spontaneous filling of the CVC lumen with blood and visible arterial pulsations. The initial CVC misplacement did not alter saturation or clinical stability.

**Fig. 1:**
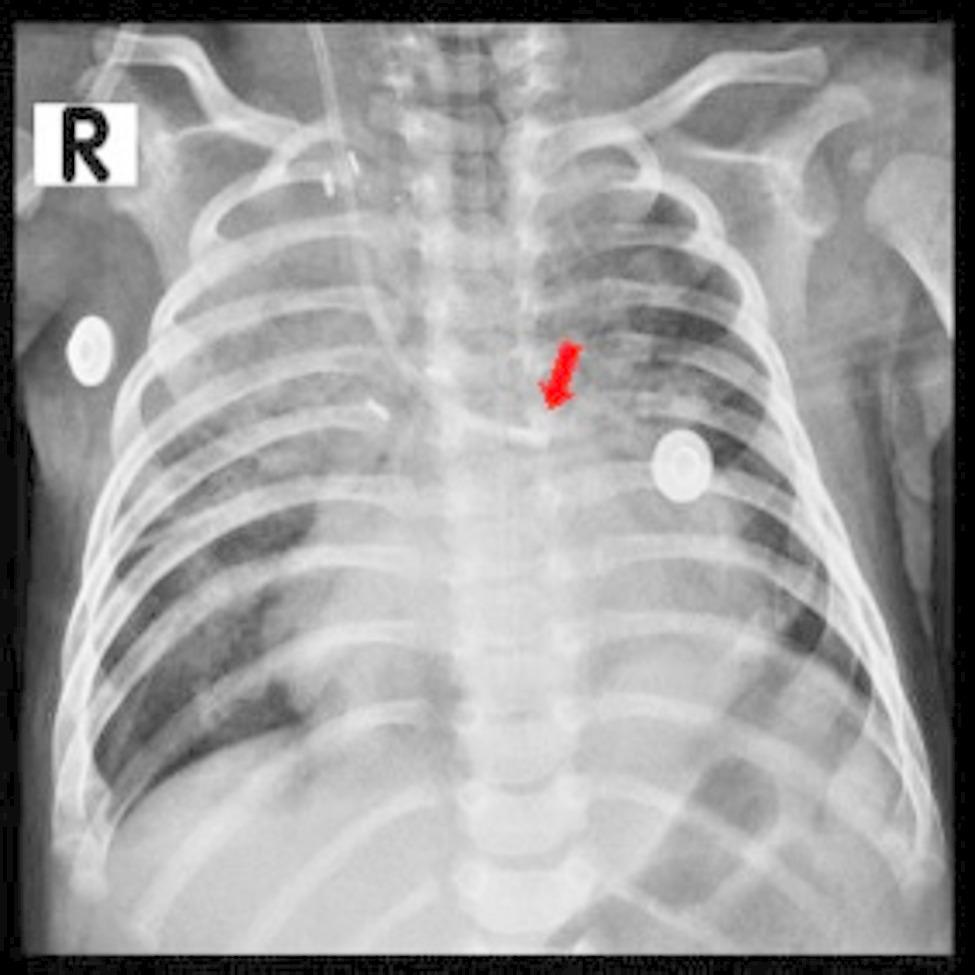
Chest x-ray performed 2 days after central venous catheter (CVC) insertion showing a left-side deviation of the inserted catheter tip (arrow). R, right

After 36 h of serum infusion through the CVC, a chest radiograph was taken, which was suspicious for the misplacement of the CVC into the pulmonary artery (Fig. 1). This repeated radiograph was obtained due to clinical suspicion (e.g., elevated SvO2, CVP) and confirmed malposition. Also, an abnormally high venous oxygen saturation (SvO2) was detected (Table 1). For further evaluation, an echocardiography was performed in which the tip of the CVC was not identified [4]. Subsequently, central venous pressure (CVP) was measured by a CVP monitoring device through CVC, which was as high as 10 to 30 mm/Hg with a mean of 15 mm/Hg. Pulmonary artery pressures were systolic: 30 mmHg; diastolic: 10 mmHg; mean: 15 mmHg.

**Table 1:** Venous blood gas test results of a sample from central venous catheter

Analyte	Test results	Normal ranges	Unit	Changes
pH	7.413	7.31–7.41	-	I
pCo2	49.6	40–52	mmHg	N
pO2	57.1	30–50	mmHg	I
HCO3-act	31.1	22–27	mEq/L	I
SvO2	89.0	65–70	Percentage (%)	I

HCO3-act: actual bicarbonate; I: increased; mEq/L: milliequivalents per litre; mmHg: millimeters of mercury; N: normal; pCo2: partial pressure of carbon dioxide;

pO2: partial pressure of oxygen; SvO2: venous oxygen saturation

**Fig. 2:**
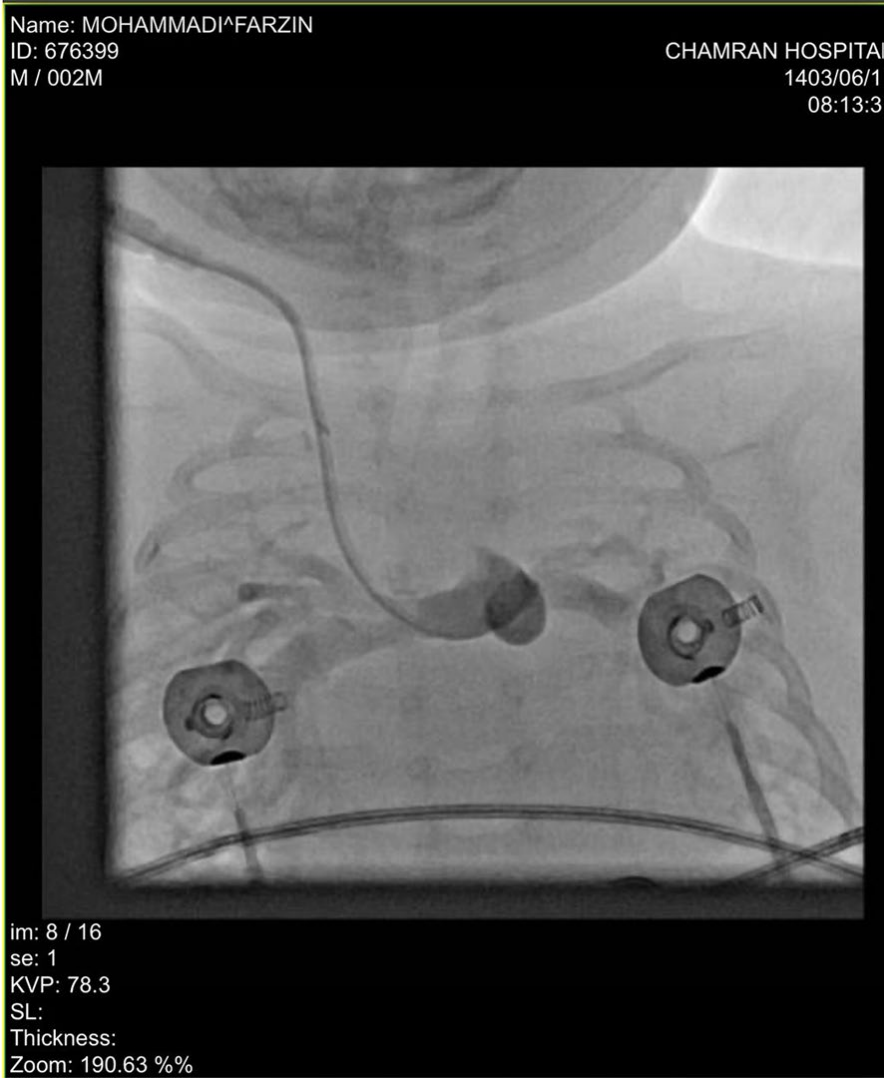
Angiogram showing opacification of the pulmonary artery after injection of contrast agent, confirming inadvertent insertion of the central venous catheter (CVC) into the pulmonary artery through the modified Blalock Taussig shunt (MBTS)

Then a cardiac catheterization confirmed the malpositioning into the pulmonary artery through the MBTS (Fig. 2). The catheter likely traversed the right carotid artery, subclavian artery, Blalock-Taussig (BT) shunt, and pulmonary artery. This pathway was confirmed via cardiac catheterization (Fig. 2). While contrast echocardiography was considered, it was deferred due to arterial route suspicion and embolism risks.

Therefore, the patient underwent a secondary procedure to remove the CVC and insert a new CVC under ultrasonography guidance. The catheter was removed under sterile conditions with manual compression to ensure hemostasis. No bleeding or hematoma occurred post-removal. Given the arterial route, the site was closely monitored post-procedure. Ultrasound guidance was reused for the second CVC placement, with meticulous attention to anatomical landmarks. Though malposition risks persist, no complications arose. The procedure was well tolerated. After completion of the patient’s treatment, he was discharged. The patient’s follow-up revealed that the treatment was successful without further complications.

## Discussion

Catheter misplacement is a rare complication of CVC insertion. Previous studies addressed arterial or extravascular misplacements of CVCs inserted into subclavian and internal jugular veins [2, 7]. This condition could result in life-threatening complications such as arterial puncture, aortic perforation, air embolism, stroke, hemothorax, and pneumothorax [6, 7]. An MBTS is the most common palliative procedure performed in children with complex CHD to help enhance blood flow to the pulmonary artery, diminishing cyanosis in children with cyanotic CHD [8]. It is anatomically positioned between the subclavian artery and the pulmonary artery, adjacent to the right internal jugular vein (IJV) at the root of the neck, where it intersects with the first part of the subclavian artery. Therefore, the right IJV, subclavian artery, pulmonary artery, and MBTS have a greater chance of being in the same needle trajectory, being more complicated by a short neck in infants where all structures are in closer proximity [9].

Here, we reported a life-threatening complication of CVC misplacement, inadvertently inserted into the pulmonary artery through the right MBTS, in a two-month-old infant with a cyanotic CHD. We suspected this complication following suspicious chest radiography, detection of an increased PvO2 drawn from CVC, and an increased CVP pressure measured through CVC and confirmed after cardiac catheterization. The likelihood of malpositioning initially seemed low in our case since the procedure was performed under ultrasonographic guidance, and no pulsatile blood flow was observed during insertion. However, a definitive diagnosis of catheter malposition was ultimately confirmed through cardiac catheterization. This was necessary as the chest radiograph alone could not provide conclusive evidence, primarily because both the innominate course and the pulmonary artery are located on the right side. The only distinguishing anatomical feature is that the innominate course lies superior to the pulmonary artery, complicating radiographic interpretation.

This complication has been previously reported in two cases [9, 10]. One of them was a neonate cyanotic at birth with pulmonary atresia, ventricular septal defect, and patent ductus arteriosus who underwent a left MBTS insertion to the distal left pulmonary artery. Following a CVC insertion into the right IJV, under the guidance of real-time ultrasound, an arterial waveform CV pressure tracing besides identical partial pressures of oxygen in concurrent venous and arterial blood gases triggered suspicions of arterial placement of the CVC. Further evaluation through a dye contrast CT scan showed a CVC going through the right IJV into the subclavian artery and then the MBTS [9]. The other case was a neonate diagnosed with pulmonary atresia and ventricular septal defect who underwent a right MBTS insertion procedure. Similar to the previous case, after a CVC insertion into the right IJV under direct real-time 2-dimensional ultrasound guidance, high mean pressures with a pulsatile waveform recorded by the neck catheter transducer and similar levels of blood gases drawn simultaneously from the right neck catheter and the radial arterial catheter made suspicion of misplacement into the arterial system, which was confirmed by a contrast-enhanced focused CT scan of the thorax [10].

Notably, our case alongside the previously mentioned cases, demonstrated that malpositioning can still occur despite the use of ultrasound guidance during the procedure. Whereas the aforementioned studies confirmed malposition using contrast-enhanced CT scans, we opted to perform cardiac catheterization for confirmation. While numerous guidelines and clinical trials advocate for the routine use of ultrasound in IJV puncture [11–13], our case, along with the previously mentioned studies, demonstrates that it is not a comprehensive solution and should be employed alongside other clinical evidence, such as manometry or pressure-waveform analysis measurements.

## Conclusion

In conclusion, CVC insertion in neonates and children with complex cardiac anatomy or with MBTS is a challenging procedure requiring extra vigilance. Due to possible variations of the venous system, prior knowledge of specific underlying anatomy is crucial in children with CHD. Additionally, it is noteworthy to mention that even though ultrasound is a useful method to reduce the risk of CVC misplacements, additional attention to clinical evidence involving manometry, pressure-waveform analysis measurements, and blood gas tests is heavily recommended. All identifiers have been removed from radiographic images to ensure patient anonymity.

## Data Availability

No datasets were generated or analysed during the current study.
